# Self-Regulation in High-Level Ice Hockey Players: An Application of the MuSt Theory

**DOI:** 10.3390/ijerph182413317

**Published:** 2021-12-17

**Authors:** Montse C. Ruiz, Reko Luojumäki, Samppa Karvinen, Laura Bortoli, Claudio Robazza

**Affiliations:** 1 Faculty of Sport and Health Sciences, University of Jyväskylä, 40014 Jyväskylä, Finland; reko.luojumaki@gmail.com (R.L.); samppa.karvinen@gmail.com (S.K.); 2 BIND-Behavioral Imaging and Neural Dynamics Center, Department of Medicine and Aging Sciences “G. d’Annunzio” University of Chieti-Pescara, 66013 Chieti, Italy; l.bortoli@unich.it (L.B.); c.robazza@unich.it (C.R.)

**Keywords:** psychobiosocial states, action components, emotion, performance, MuSt theory

## Abstract

The purpose of the study was to examine the validity of core action elements and feeling states in ice hockey players in the prediction of performance. A second aim of the study was to explore the effectiveness of a 30-day program targeting action and emotion regulation. Participants were male ice hockey players drawn from two teams competing at the highest level of the junior Finnish ice hockey league. They were assigned to a self-regulation (*n* = 24) and a control (*n* = 19) group. The self-regulation program focused on the recreation of optimal execution of core action elements and functional feeling states. Separate repeated measures MANOVAs indicated significant differences in ratings of perceived control and execution accuracy ratings of self-selected visual and behavioral components of the action (critical for optimal performance) and psychobiosocial (feeling) states across recalled best and worst games. Results support the use of both action- and emotion-centered strategies for performance enhancement. Future research including psychophysiological markers is warranted.

## 1. Introduction

Substantial literature indicates that athletes’ subjective experiences influence their performance [1,2,3,4,5], and that they use several strategies to regulate emotions in order to attain sporting achievements [6,7]. Emotion regulation refers to the processes by which individuals manage the type, intensity, or duration of emotions they experience [8]. However, emotion regulation is only one part of the self-regulatory process by which individuals modify or modulate their thoughts, feelings, and behaviors to attain goals [9,10]. Thus, intervention programs aimed to help athletes reach and maintain optimal performance that combine emotion- and action-centered regulation strategies are assumed to be more effective than focusing on each of them in isolation [9,11].

A novel theoretical framework that considers both emotion- and action-regulation is the multi-states (MuSt) theory [1]. Similar to previous theoretical frameworks [12,13], the MuSt theory acknowledges the importance of athletes’ evaluation of their interaction with the environment, which determines their experiences and the perceived functional impact on performances. Extending previous frameworks, the MuSt theory accounts for the multiple performance states of athletes which derive from the interaction between valence (pleasant–unpleasant) and functionality (functional–dysfunctional) of their subjective experiences, and the level of attention monitoring/control they exert on task execution. High level performance can be characterized by a pleasant experience, and a low level of action monitoring, leading to ‘flow’ states [14]. High level performance can also be characterized by unpleasant experiences and high action monitoring, a more effortful ‘clutch’ state than flow [15]. In this case, directing one’s attention only to relevant key elements of the action may help prevent excessive conscious control of movement, or a wrong focus of attention. Poor performance is typically typified by focus on irrelevant aspects, lack of energy, and dysfunctional states.

Self-regulation is a process that involves awareness, acceptance, and goal-directed actions [9,16]. A requisite of successful regulation involves athletes’ awareness of the type of experiences they have, and how these may influence their performance. Athletes’ experiences are usually characterized by several interrelated aspects, including psychological (i.e., affective, cognitive, motivational, and volitional), biological (i.e., bodily and motor–behavioral), and social (i.e., operational and communicative) components, which together form the so-called psychobiosocial states [17]. According to the MuSt theory, functional psychobiosocial states (pleasant or unpleasant) are those that are energizing, and reflect a task-relevant focus or effortless action monitoring. Conversely, dysfunctional psychobiosocial states (pleasant or unpleasant) are those that reflect a lack of energy, or an athlete’s struggle to channel energy appropriately, thus, resulting in distraction from the task at hand, or excessive conscious control over processes that would require little attention. This multi-component conceptualization of athletes’ feeling states has been supported in several studies involving athletes of different sports and competitive levels [4].

Athletes’ acceptance of their experiences is the next necessary step prior to the implementation of regulation strategies. Awareness and acceptance are also common core elements in other intervention modalities, such as the mindfulness–acceptance-based approaches [18,19]. However, though these approaches do not seek to change the content of athletes’ experiences [19], self-regulation involves deliberate behaviors aimed to down-regulate dysfunctional experiences, or up-regulate or maintain functional experiences [9]. Some of the strategies that athletes can use to regulate their emotions include relaxation (e.g., focus on breathing), imagery [20], music [21,22], or mindfulness–acceptance-based approaches [23]. Based on these self-regulation strategies the athletes often use spontaneously, during the intervention, we combined somatic techniques (i.e., paced breathing and relaxation) and mental imagery of challenging competitive situations to help players experience and mentally strengthen the optimal conditions for performance. Deep, rhythmic, abdominal breathing at approximately six breaths per minute is recommended to increase heart rate variability (HRV), which refers to changes in the time intervals between heartbeats. When individuals face stressful events in high HRV conditions, they are better able to respond, react, and adapt [24]. Thus, HRV training has been used to increase the athlete’s ability to self-regulate and, consequently, to improve sport performance [25]. Additional benefits associated with HRV training may come from greater self-awareness of physical and mental states, as well as a better understanding of the mind–body connection [26].

Beyond regulation of their own experiences for successful performance, athletes are also required to shift the focus of attention appropriately from internal feelings to task execution [27]. In line with MuSt theory assumptions, a task-focused attentional monitoring underlies automatic processes, and is associated with good performance, whereas an excessive self-focused attention results in controlled processes and impaired performances.

### Study Purpose and Hypotheses

Framed within the MuSt theory [1], and following the principles of self-regulation [9], the purpose of this study was to examine the effectiveness of an intervention program on the self-regulation of core action components and psychobiosocial states of ice hockey players. We also examined the predictive validity of a whole profile including action elements and feeling states. We hypothesized that core action components and psychobiosocial states would differentiate between best and worst performances (H1). We also expected that the intervention would be effective in helping players self-regulate their core action components and psychobiosocial states (H2), which would be reflected in improved perceived performance (H3).

## 2. Materials and Methods

### 2.1. Participants

A priori power analysis for multivariate analysis of variance (MANOVA) design (within-subjects main effect), with an anticipated medium effect size (f = 0.30), statistical power set at 0.80, α level of 0.05 (correlation among repeated measures = 0.5), and three measurements, suggested a minimum sample size of 22 (G*Power 3.1.9.6 software) [28]. We recruited 43 Finnish male ice hockey players with a mean age of 19.98 years (SD = 0.95). The criterion for participation in the study was having a minimum of 8 years of playing experience. Participants were drawn from two teams competing at the highest level of the Finnish A-junior ice hockey league. Their playing experience ranged from 8 to 18 years (*M* = 13.70, *SD* = 2.18). The participants were non-smokers, did not report any sleep disturbances, heart or respiratory conditions, and had not previously participated in any structured self-regulation interventions.

### 2.2. Measures

#### 2.2.1. Core Action Components

Players identified two visual and two behavioral core components of the action (ice hockey task-execution) following a stepwise profiling procedure [11]. First, players were asked to identify a challenging situation occurring during game where their performance would be likely subjected to high variability. The key question was: “*Imagine yourself performing in a mental or physical nonoptimal state, for example when you are under distress or fatigue, or after a mistake or a poor execution, or when you have to deal with unexpected events. What are the actions or behaviours that you would need to control intentionally in order to execute in a consistent and accurate manner, and thus attain good performance?”* For example, a player may identify several elements within the faceoff situation (e.g., going to the circle, opponent’s positioning, opponent’s blade, own teammates positioning, own positioning, grip of the stick, own blade’s angle, reaction to the puck drop). Participants were then asked to identify two most important visual cues (e.g., opponent’s positioning, opponent’s blade) and two behavioral cues (e.g., own positioning, own blade’s angle) of the action that would be most important to regulate. Then, they were asked to assess the perceived accuracy and control levels of execution of each component on the modified Borg scale [29]. To avoid floor and ceiling effects, the following verbal anchors were used: 0 (*nothing at all)*, 0.5 (*very, very little)*, 1 (*very little),* 2 (*little)*, 3 (*moderate*), 5 (*much),* 7 (*very much)*, 10 (*very, very much)*, and • (*maximal possible, 11 score is attributed to maximal possible*).

#### 2.2.2. Psychobiosocial States

Individualized Profiling of Psychobiosocial States (IPPS) [17] was used to assess several aspects of the players’ performance related experiences (i.e., emotional, cognitive, motivational, volitional, bodily, motor–behavioral, operational, and communicative). The IPPS consists of 20 rows of items, including in total 74 descriptors (3–4 per row). Functionality (functional, dysfunctional) and hedonic tone (pleasant, unpleasant) distinctions were used in the development of the items assessing the emotional modality, which is assessed on six items (i.e., functional pleasant states, dysfunctional pleasant states, functional anxiety, dysfunctional anxiety, functional anger, and dysfunctional anger). The functionality distinction alone was used to categorize the items assessing cognitive, motivational, volitional, bodily, motor–behavioral, operational, and communicative aspects of the psychobiosocial states. For example, the bodily modality is assessed on two items, one describing functional bodily aspects (e.g., vigorous, energetic, physically charged), and one describing dysfunctional bodily aspects (e.g., physically tense, jittery, tired, exhausted). Participants are asked to select one adjective per item to describe how they are feeling. Then, they rate the intensity of their states on a scale ranging from 0 (*nothing at all*) to 4 (*very much*). High reliability and sound factorial structure for a two-factor solution (functional, dysfunctional) in a sample of Finnish athletes has been reported [30].

#### 2.2.3. Imagery Ability

Participants’ imagery ability was measured on the Sport Imagery Ability Measure (SIAM) [31]. The 48-item SIAM consists of four sport-related scenarios. Participants receive detailed descriptions of each scenario, after which they are given one minute to imagine each scenario. Then, they are asked to complete twelve items to assess their imagery on five dimensions (i.e., vividness, control, duration, ease, and speed of generation), six senses (i.e., visual, auditory, olfactory, gustatory, tactile, and kinesthetic), and the experience of emotion. Each item (e.g., “How clear was the image”) is rated on a 100 mm visual analogue scale (e.g., “*no image*” to “*perfectly clear image*”). Adequate internal reliability and sound factorial validity for hard copy and web-based versions of the SIAM have been reported [32].

#### 2.2.4. Heart Rate Variability

Cardiac vagal activity was measured as beat-to-beat intervals using the Firstbeat Bodyguard 2 (Firstbeat Technologies Ltd. Jyväskylä, Finland). The device is attached to the body with two built-in electrodes, one to be placed under the collarbone, and the second one on the rib cage. The sampling frequency is 12.5 Hz. Measurement starts automatically when the device is attached. Very high beat detection rate (i.e., 99.95%) has been reported [33]. The root mean square of successive differences (RMSSD) was used as reliable and stable measure of HRV [34,35].

#### 2.2.5. Performance

Perceived overall performance was measured on the modified Borg scale [29]. The range was similar to the assessment of core elements; however, only two verbal anchors were included, thus, perceived performance ranged from 0 (*extremely poor)* to • (*excellent).* A score of 11 was attributed to excellent. In addition, participants’ actual ice hockey performance was video recorded from a bird’s eye perspective, where half of the performance platform is visible at a time, with a dynamic center to the puck (broadcast view of the ice hockey rink).

#### 2.2.6. Feedback and Evaluation

A brief semi-structured interview was conducted to assess participants’ involvement and evaluation of the study. Participants were asked to reflect about their involvement in the study, frequency and easiness following the breathing and imagery instructions, and overall perception of the program. The purpose of the interview was to serve as a manipulation check to determine the compliance with the intervention proposed, and to capture participants’ experiences about the self-regulation program.

### 2.3. Procedure

Ethical approval for the study was granted from the local institution’s ethics committee. The study was conducted in accordance with the Declaration of Helsinki. Managers and coaches of two ice hockey teams were contacted, and the purpose of the study was explained to them, after which they granted permission to contact the participants. Participants were informed about the purpose of the study, procedures, confidentiality of results, and voluntary nature of study participation. They signed an informed consent form prior to commencement of the study, and were requested to create a code to ensure anonymity.

**Phase one: Recalled game situations.** This phase consisted of the initial assessments of core components of the action, psychobiosocial states, and imagery ability, as well as the description of the study procedures. The assessments were carried out individually or in small groups in a quiet place nearby training facilities. Specifically, participants were requested to identify the most relevant core components of the action. The players were asked to access video recordings via personal electronic devices to identify their best game. They could watch the whole game or shorter individualized performance situations as many times as they wanted. After watching video recordings, participants were asked to assess the level of control and perceived accuracy of the previously selected core components of the action, as well as to identify and rate the intensity level of psychobiosocial states when thinking of their best performance. Previous research has shown that video-assisted recall is useful for an accurate assessment of emotions related to performance [36]. Then, participants assessed their core components of the action and how they felt in relation to their recalled worst game. Moreover, the players assessed their imagery ability on the SIAM. Finally, they were instructed about how to attach Firstbeat Bodyguard 2 chest belts to themselves. They were also requested to wear the devices for two days, to follow their normal sleep routine, and to record their sleep and waking time. Initial assessment took place a few weeks after the season had started to ensure that participants had experience and awareness of relevant aspects of their performance and feelings.

**Phase two: Actual game situations.** Participants were contacted before an actual game. They were asked to wear the Firstbeat Bodyguard 2 chest belts, and to record their HRV during the two days prior to the game. A day after the game, with the aid of video-assisted recall, the players were asked to assess the level of control and perceived accuracy of their core action components and psychobiosocial states. They also assessed their perceived overall game performance.

**Phase three: Paced breathing and self-regulation protocols.** Participants from one team were assigned to a self-regulation condition (SR group), whereas participants from the second team were assigned to a control condition (C group). SR group players were instructed to follow a video including a breathing pacer and guidelines for slow-paced breathing (6 cycles/min; 4.5 s inhale and 5.5 s exhale) for 15 min. At the end of the 15 min, they received the following instructions:

*“Now you can breathe at your own pace. Imagine the challenging situation you have previously identified. You may close your eyes if you feel more comfortable. Imagine yourself performing optimally in your challenging situation. While performing optimally in your challenging situation, pay attention to your core action components. Imagine yourself performing these core components accurately. You feel in control of the situation. Experience the emotions and feelings you felt when you have performed your challenging situation optimally. Pay attention to how you feel yourself, your muscles, and your whole body. Now, you can continue imagining your core components, the emotions, and experiences you had while performing your challenging situation optimally.”*

C group players were instructed to follow a video including a breathing pacer and guidelines for a close to normal breathing rhythm (12 cycles/min; 2.2 s inhale and 2.8 s exhale) for 15 min. At the end of the 15 min, they received the following instructions:

*“Now you can breathe at your own pace. Imagine yourself at the ice rink… You may close your eyes if you feel more comfortable. Now, imagine yourself executing your usual routine in preparation for a practice session. You put on your hockey equipment, shin guards, socks, pants, skates, shoulder pads, elbow pads, jersey, helmet, globes … Focus on how the equipment feels like, what you see around, who is there with you. Once you are ready, imagine you warm-up to prepare for a normal practice session.”*

SR and C group players were asked to follow the video instructions (paced breathing and imagery) for one month. Daily reminders were sent to all participants inviting them to follow the instructions. HRV, ratings about self-selected action elements, and feeling states were assessed in relation to actual games and post-intervention. Specifically, all participants measured their HRV (for the two consecutive days prior to the game). With the aid of video recordings, they also assessed their core action components, psychobiosocial states, and perceived performance (after the game).

**Phase four: Evaluation and feedback.** Finally, one/two weeks after the intervention, participants were individually interviewed inquiring about their involvement in the study. They were asked to provide information about compliance with the program, and to reflect on the perceived effectiveness.

### 2.4. Data Analysis

Data was screened for missing values, distribution, and possible outliers. A series of repeated measures MANOVAs was used to investigate the differences on the dependent variables (core action components, psychobiosocial states, performance, HRV). The independent variables were group (SR group vs. C group), context (best performance vs. worst performance), and time (pre-, post-intervention). Pair-wise comparisons of means were then calculated to ascertain sources of significant effects.

## 3. Results

**Figure 1:**
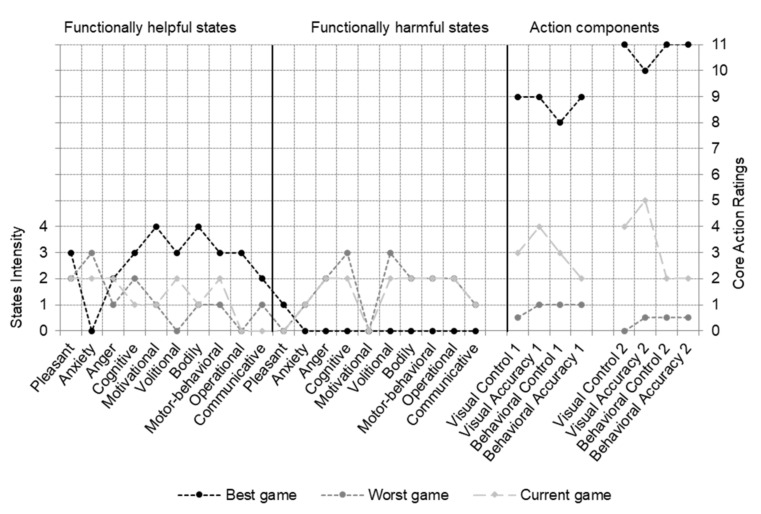
Ice hockey player’s individual profile of psychobiosocial states and core components of the action in best, worst, and current games.

Three cases had missing data (missingness > 5%), and were thus deleted. An individual profile including ratings for the self-selected core action elements and intensities of feeling states is depicted in Figure 1. Descriptive statistics for core action elements and feeling states identified in recalled best and worst games at group level is presented in Table 1.

As expected, significant differences were found in the ratings of the action components across context (recalled best and worst games), Wilks’ λ = 0.071, *F*(8, 34) = 55.463, *p* < 0.001, η*_p_*^2^ = 0.929. Post hoc analysis indicated that there were significant differences in all action elements (*p* < 0.001, η*_p_*^2^ values ranged from 0.630 to 0.888). There were no significant differences in the ratings of action components in recalled best and worst games across groups.

Significant differences were found in the intensities of psychobiosocial states across recalled best and worst games, Wilks’ λ = 0.098, *F*(20, 20) = 9.227, *p* < 0.001, η*_p_*^2^ = 0.902. Post hoc analysis indicated that there were significant differences in all modalities of psychobiosocial states (*p* < 0.001, η*_p_*^2^ values ranged from 0.382 to 0.759), excluding the Pleasant (−) modality. There were no significant differences in the intensities of feeling states across groups.

There were no significant differences across SR and C group players in the ratings of selected action elements at pre-intervention assessment in an actual game, Wilks’ λ = 0.652, *F*(8, 24) = 1.604, *p* = 0.176, η*_p_*^2^ = 0.348. Similarly, no significant differences emerged in the intensities of psychobiosocial states at the baseline actual game assessment, Wilks’ λ = 0.472, *F*(20, 12) = 0.670, *p* = 0.793, η*_p_*^2^ = 0.528.

**Figure 2:**
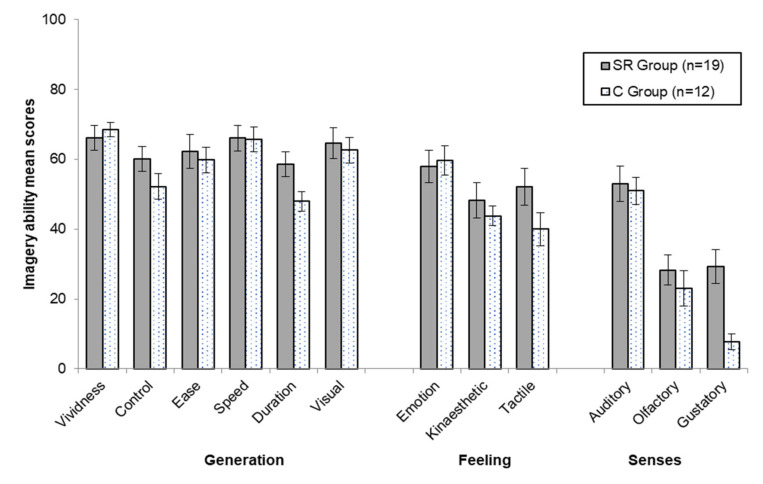
Mean and standard deviations of ice hockey players’ imagery ability subscale scores at phase one.

Participants’ mean scores in the imagery ability subscales at phase one (pre-intervention) are depicted in Figure 2. There were no significant differences in the imagery ability subscale scores across SR and C group players, Wilks’ λ = 0.483, *F*(12, 18) = 1.604, *p* = 0.107, η*_p_*^2^ = 0.517. Their imagery ability was deemed sufficient to follow the study protocols.

**Figure 3:**
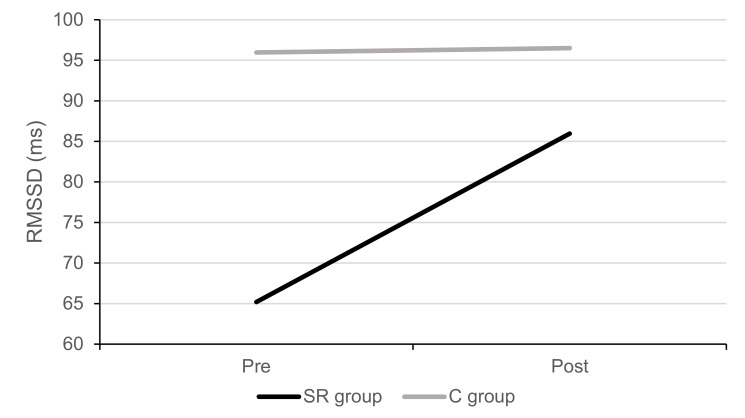
Root mean square of successive differences between RR intervals (RMSSD) for heart rate variability during sleep at the beginning and the end of the training program.

Repeated measures MANOVAs yielded significant differences by group for RMSSD mean scores recorded during sleep at pre- and post-training assessments, Wilks’ λ = 0.542, *F*(2, 15) = 6.345, *p* = 0.010, η*_p_*^2^ = 0.458. Post hoc analysis indicated significant differences in the RMSSD values prior to the commencement of the training program, with a significant increase in variability for SR participants (see also Figure 3).

MANOVA could not be calculated to examine differences in the ratings of the action components and intensities of feeling states at pre- and post-intervention across groups due to the high attrition, with 19 participants providing data at both times. Separate independent samples Mann–Whitney U tests indicated that there were no significant differences by groups in the ratings of the action components (*p* values > 0.289) or in the intensities of psychobiosocial states (*p* > 0.056) across pre-and post-intervention.

Contrary to our third hypothesis, independent samples Mann–Whitney U tests did not yield significant differences by groups in the ratings of perceived overall performance in actual games played pre- and post-intervention (*p* = 0.643).

Participants reported high compliance with the program. SR group participants reported following the video instructions three to seven times per week (*M* = 5.00; *SD* = 1.44), whereas C group participants followed them from two to seven times per week (*M* = 5.00; *SD* = 1.86). Overall, players perceived the program to be beneficial, rating its benefit on average 3.77 (*SD* = 0.49), with 5 being highest rating, and an average of 3.67 (SD = 0.61) for participants in the control group.

Qualitative feedback indicated that the self-regulation program was beneficial. For example, “I noticed that the more I practiced the better it went, it was more natural, and I calmed down and felt more relaxed. I got such a good and relaxed feeling, that... I could have done anything after that.” “I relaxed. It’s not often in everyday life that you can relax like that, in such a comprehensive way. If the day was a bit hectic, this was incredibly helpful.”

The participants also reported their involvement in the study helped them gain awareness, as the following quotes illustrate: “I hadn’t thought so much about how one can influence his own feelings. So now that I think about it, the feelings are different when it goes well, compared to when goes badly. If it goes well, then you are inside the game. If you are not absorbed in the game, then the focus goes away, you are not focused on the puck…” “Thinking about your feelings, and after a situation that is very important, thinking about your feelings, because they have big influence in that, and then you are able to modify and change for the next situation.” They also reported the program was helpful to self-regulate in game situations, for example: “when it is not going well in a game, then you can reset, take a few deep breaths” “Especially if there is a bad performance cycle then you can break it into smaller parts, and if the performance is good, then you have to find out what worked, and continue from there.”

## 4. Discussion

Drawing from MuSt theory assumptions [1] and principles of self-regulation [9], this study examined the validity of using core action elements and feeling states in the prediction of performance in high-level ice-hockey players. A second purpose of the study was to examine the effectiveness of a 30-day intervention program targeting the regulation of players’ action and psychobiosocial feeling states. Overall, our findings support the feasibility of action and feeling states self-regulation programs within high level performance. The findings also provide support for theoretical assumptions and principles of self-regulation extending the previous literature on athletes’ action and feeling states regulation.

In line with our first hypothesis, the results indicated that the players identified two clearly distinct profiles of core action elements and feelings states associated with their most successful versus least successful games. All players were able to identify core visual and behavioral elements of their action and feeling states associated with their most and least successful games. With respect to their best performances, the players reported high control and accuracy of the visual and behavioral elements, as well as high intensities of functional states, and low intensities of dysfunctional states. The findings showed the opposite for least successful games, that is, low accuracy and control of the visual and behavioral elements, high intensities of dysfunctional states, and low intensities of functional states. Previous research examining self-identified core action elements targeted athletes from an individual and self-paced sport (i.e., shooting) [11,13]; thus, findings extend the study of core action elements and feeling states to team sports in a naturalistic setting.

Video recorded performances were used to facilitate the assessment of players’ action elements, and to help participants recall their experiences. Video recordings, commonly available in high level teams, have been successfully used in previous studies examining emotions and behavioral components of emotions in naturalistic settings [36,37]. This methodology is helpful when assessing athletes’ experiences or performance during competitions. In addition, the use of video-assisted recall may help minimize the potential influence of the results in the assessments of players’ experiences.

The results on the action elements and experiences associated with best and worst performances are in line with previous research examining peak performance experiences of athletes, which are typified by clear focus on the task, and a balance between their skills and task demands [38]. However, our findings also suggest that focusing on core components of the action can help athletes attain good performance even when they experience nonoptimal states. This result concurs with assumptions of the MuSt theory [1] and other approaches, such as the multi-action plan model [11] or the integrated model of flow and clutch states [15].

The 30-day self-regulation intervention program included elements from imagery, and slow-paced breathing. A priori assessment of the players’ imagery ability indicated a moderately high level regarding image generation (i.e., vividness, control, ease, speed, duration, visual), and aspects related to diffuse body feeling states (i.e., emotion, kinesthetic, tactile) associated with the creation of mental representations in sport settings (Figure 2). Lower scores were reported for single senses (i.e., auditory, olfactory, gustatory). These scores are similar to those reported by athletes from a wide range of sports, including team sport athletes competing at high level, who also reported much lower scores for the gustatory and olfactory aspects of imagery [32].

Compared to participants in the control group instructed to follow a breathing pattern akin to spontaneous breathing, a significant increase in vagal tone was observed for the participants intervention group after the 30-day program. This finding is in line with previous research suggesting that slow-paced breathing can improve sleep quality and cardiac vagal activity in non-athletic populations [39].

Contrary to our second hypothesis, our results did not yield significant differences across SR and C groups in the accuracy and control ratings of the selected action elements, or in the intensities of the feeling states associated with actual performances. In addition, we did not observe significant differences in perceived overall performances across actual games across pre- and post-intervention (hypothesis 3). In explaining this lack of significant differences, we may speculate that the repeated assessments of the aspects related to the action and experiences of the participants could have increased the awareness in all participants. Though participants in the control group were not given specific self-regulation instructions, they may have employed their own strategies to attempt to self-regulate. The creation of individual profiles comprising action components and psychobiosocial states can stimulate reflection on the conditions, leading to successful and unsuccessful performances, increase the awareness of functional and dysfunctional states, and, therefore, lead the athlete to identify and initiate self-regulation strategies [40,41]. In addition, with ice hockey being a reactive team sport, overall performance not only depends on individual players, but also depends on the performance of teammates and opponents, which may have influenced participants’ ratings. Qualitative accounts for participants’ involvement in the self-regulation program indicated that they perceived it as beneficial for self-regulation. This finding concurs with the view that individual appraisal of teammates’ expressions of feelings in response to constantly changing events in a competition can suddenly elicit emotional, cognitive, and behavioral responses in other players, which can facilitate social interactions, as well as individual and team performance [42].

### Strengths, Limitations, and Future Research Directions

A strength of this study is that it was conducted in a naturalistic setting involving high level ice-hockey players. Core action components and experiences were measured in relation to actual game situations, thus, increasing the ecological validity of the findings. Secondly, and in line with the multi-modal conceptualization of athletes’ psychobiosocial experiences, the intervention involved cognitive and somatic strategies, such as slow-paced breathing, aimed to create and recover optimal experiences, and facilitate mental rehearsal of core action elements.

Despite the meaningful findings, this study has some limitations that need to be considered. The first limitation is related to participant attrition, which did not allow for group comparisons using parametric statistical analysis. However, the qualitative data collected gave an insight into the players’ experiences. Further research is needed to further examine our study hypotheses, also extending the investigation to female athletes. Secondly, similarly to the players in the experimental group, participants in the placebo-control group assessed accuracy and control of their action components, as well as their experiences, which may have influenced the results. Thus, study designs where participants are randomly assigned to a control group involved in tasks completely different from those included in the intervention may provide more clear results. Finally, participants in this study did not receive immediate feedback on their HRV data to avoid contamination of results. Future research using biofeedback training can examine the effectiveness of providing immediate feedback regarding HRV to help participants self-regulate.

## 5. Conclusions

The purpose of this study was to examine the predictive validity of a whole profile including core elements of the action and feelings states in high-level ice hockey players. As expected, the players identified two very distinct profiles of core action elements and feelings for their best performances and worst performances. Specifically, profiles of best performances reflected high control and accuracy of visual and behavioral elements, and high intensities of functional states. Profiles of worst performances reflected low accuracy and control of visual and behavioral elements, and high intensities of dysfunctional feeling states.

A 30-day self-regulation intervention including imagery and slow-paced breathing was effective in increasing players’ vagal tone, reflecting higher HRV during sleep. Contrary to our hypothesis, no significant differences were found in accuracy or control ratings of visual and behavioral elements, or in the intensities of feeling states in actual games. Players’ perceptions about the intervention indicated that the self-regulation program was beneficial, helping players increase their awareness. Overall, the findings provide support for the combination of strategies targeting the regulation of core action elements and feeling states.

## Figures and Tables

**Table 1:** Descriptive statistics for players’ psychobiosocial states and core action components in recalled best and worst games for participants in the self-regulation and control groups.

	Self-Regulation Group (*n* = 24)								Control Group (*n* = 19)							
	Best Games				Worst Games				Best Games				Worst Games			
Variables	*M*	*SD*	*SK*	*K*	*M*	*SD*	*SK*	*K*	*M*	*SD*	*SK*	*K*	*M*	*SD*	*SK*	*K*
Core action components																
Visual 1 Control	7.46	2.26	−1.17	0.62	3.83	3.01	0.70	−0.43	6.95	2.34	−0.74	−1.06	2.32	1.10	−0.32	−0.95
Visual 1 Accuracy	8.08	1.84	−1.73	4.23	2.96	2.27	0.68	−0.53	8.26	1.59	−2.05	6.12	3.05	2.20	1.65	3.02
Behavioral 1 Control	7.96	2.07	−0.96	0.51	3.08	2.50	0.79	−0.22	8.53	1.39	−2.08	5.71	4.21	2.46	0.43	0.06
Behavioral 1 Accuracy	8.58	1.91	−2.08	5.57	2.63	2.22	0.95	−0.03	8.68	1.25	−1.62	3.20	2.58	1.38	−0.18	−1.13
Visual 2 Control	7.69	2.62	−1.32	1.11	3.96	3.18	0.53	−1.07	7.58	2.29	−0.44	−0.99	2.53	1.43	−0.31	−0.69
Visual 2 Accuracy	8.04	2.10	−2.13	4.97	2.92	2.61	1.11	1.01	8.58	1.68	−1.06	0.15	1.84	1.05	0.15	−0.51
Behavioral 2 Control	8.75	1.65	−1.15	1.64	3.10	2.56	1.12	0.56	8.11	2.62	−1.76	4.16	3.42	2.41	1.44	2.24
Behavioral 2 Accuracy	8.79	1.56	−1.87	4.02	2.21	1.50	0.66	−0.53	8.32	2.71	−1.97	4.21	1.89	1.23	0.10	−0.85
Psychobiosocial States																
Pleasant (+)	3.50	0.59	−0.69	−0.40	1.42	1.21	0.37	−0.86	3.53	0.51	−0.11	−2.24	1.63	1.01	0.50	0.42
Anxiety (+)	1.25	0.99	0.04	−1.15	2.38	1.10	−0.19	−0.49	1.16	0.83	−0.32	−1.49	2.42	1.07	0.23	−1.10
Anger (+)	3.00	0.83	−0.49	−0.17	1.83	1.17	0.53	−0.43	2.74	1.15	−0.90	0.37	2.00	1.05	0.00	−0.81
Cognitive (+)	3.25	0.94	−1.90	5.09	1.25	1.15	0.77	−0.10	3.11	0.66	−0.11	−0.39	1.11	0.88	0.34	−0.46
Motivational (+)	3.13	1.08	−1.41	1.92	1.58	1.18	0.04	−0.82	3.37	0.60	−0.31	−0.55	1.74	1.10	0.59	−0.84
Volitional (+)	3.21	0.83	−1.41	2.56	1.35	1.27	0.89	−0.16	3.05	0.62	−0.03	0.02	1.37	1.21	0.66	−0.43
Bodily (+)	2.96	1.04	−1.17	1.56	1.00	1.06	0.71	−0.70	3.00	1.00	−0.75	−0.31	1.84	1.07	−0.27	−1.27
Motor–behavioral (+)	2.96	0.86	−0.37	−0.53	1.50	1.14	0.00	−1.39	3.05	0.91	−0.60	−0.39	1.47	1.12	0.47	−0.11
Operational (+)	3.04	0.95	−1.40	3.16	1.13	1.19	0.75	−0.28	2.89	0.81	−0.50	0.30	1.32	1.11	0.66	0.35
Communicative (+)	2.13	1.19	−0.43	−0.70	0.96	1.04	0.84	−0.36	1.95	0.85	−0.50	−0.01	1.26	1.19	0.74	−0.11
Pleasant (−)	1.50	1.22	0.32	−0.97	1.04	1.23	0.98	−0.11	1.37	1.26	0.71	−0.64	0.84	1.01	0.70	−0.96
Anxiety (−)	0.46	0.78	1.96	4.02	1.87	1.18	0.46	−0.50	0.53	0.70	1.00	−0.09	1.79	1.18	0.45	−0.50
Anger (−)	0.25	0.53	2.13	4.14	1.79	1.28	0.02	−1.07	0.89	0.74	0.17	−1.00	2.26	0.87	−0.01	−0.70
Cognitive (−)	0.75	1.15	1.65	2.01	2.83	0.96	−0.28	−0.88	0.58	0.69	0.81	−0.37	2.63	0.83	−0.47	0.04
Motivational (−)	0.25	0.85	4.16	18.34	1.13	1.15	0.48	−1.26	0.11	0.32	2.71	5.98	1.26	1.10	0.82	0.68
Volitional (−)	0.17	0.38	1.91	1.79	2.04	1.30	0.05	−1.03	0.42	0.51	0.35	−2.11	2.26	0.93	−0.13	−1.10
Bodily (−)	0.79	1.06	1.64	2.71	2.78	1.17	−0.67	−0.23	1.05	0.78	0.69	0.98	2.89	0.99	−0.53	−0.61
Motor–behavioral (−)	0.46	0.93	2.07	3.33	2.38	1.06	−0.12	−0.18	0.21	0.54	2.66	6.88	2.05	0.85	−0.72	0.37
Operational (−)	0.21	0.41	1.53	0.38	2.04	1.55	−0.08	−1.46	0.16	0.50	3.34	11.19	2.11	0.88	−0.22	1.46
Communicative (−)	0.38	0.71	2.46	7.34	1.50	1.41	0.60	−0.99	0.32	0.58	1.77	2.54	1.74	0.81	−0.17	−0.16

Note. (+) denotes functional modalities of psychobiosocial states, (−) denotes dysfunctional modalities of psychobiosocial states.

## Data Availability

Data sharing not applicable.
